# Supplementary material for: Impact of diabetes mellitus on postoperative outcomes in individuals with non-small-cell lung cancer: A retrospective cohort study
Source: PLoS One. 2020 Nov 9;15(11):e0241930. doi: 10.1371/journal.pone.0241930 (PMC7652320; doi:10.1371/journal.pone.0241930)
Supplement: S1 Table — (DOCX) [file pone.0241930.s001.docx]

Supplementary Table

The included studies regarding the effect of DM on survivals in patients managed surgically for NSCLC

| Study | Country | Detailed data | Conclusions |
| --- | --- | --- | --- |
| Rezaei Hachesu, et al. (2017) | Iran | High relative impact score of DM on one-year postoperative mortality | Worse one-year survival |
| Washington, et al. (2013) | U.S.A. | 5-year survival (DM vs without);  43% vs. 47%  HR 1.08, p = 0.63 | No effect on  5-year survival |
| Bartling, et al. (2011) | Germany | 20-month survival (DM vs without);  76% vs. 59%, p = 0.04  60-month survival; 35% vs. 32% | Better 20-months survival  Worse 60-month survival |
| Dhillon, et al. (2014) | U.S.A. | 5-year survival (DM vs without);  55% vs. 50%, p = 0.74 | No effect on overall survival |
| Deng, et al. (2019) | China | Meta-analysis (13 cohort studies)  (DM vs without);  HR; 1.30, 95% CI; 1.05-1.60  p = 0.016 | Worse overall survival |
| Motoishi, et al. (2018) | Japan | 5-year survival (DM vs without);  62% versus 74%, p = 0.34 | No effect on overall survival |
