# Supplementary material for: Impact of diabetes mellitus on postoperative outcomes in individuals with non-small-cell lung cancer: A retrospective cohort study
Source: PLoS One. 2020 Nov 9;15(11):e0241930. doi: 10.1371/journal.pone.0241930 (PMC7652320; doi:10.1371/journal.pone.0241930)
Supplement: Attachment — Submitted filename: Rebuttal letter for resubmission (final PLOS One).docx [file pone.0241930.s003.docx]

September 29, 2020

Dr. Joerg Heber,

Editor in chief,

*PLOS ONE*

Dear Dr. Joerg Heber:

Thank you very much for your e-mail and review of the manuscript (PONE-D-20-19563) that we submitted on June 25, 2020. We thank the editor and the reviewer for providing constructive comments that have significantly improved the quality of the original manuscript.

**Response to the editor’s comments:**

1. The editor has raised important and interesting points regarding medications for DM.

Owing to the limitation of data retrieval, we could not identify which patients with diabetes were treated with insulin. Therefore, we were unable to evaluate the difference in 5-year survival rates between patients who were treated with or without insulin.

2. The editor has pointed out that insulin may be associated with the progression of non-small cell lung cancer (NSCLC). This possibility has recently been discussed in the literature. As mentioned above, we are unable to evaluate whether insulin might have influenced NSCLC growth owing to the limitation of data retrieval. We think that analyses regarding the association between insulin and NSCLC growth is really interesting and needed.
3. In the present study, a variety of antihyperglycemic medications were prescribed for diabetes, such as metformin, sulfonylureas, DPP-4 inhibitors, thiazolidinediones, and glucagon-like peptide agonists. These were prescribed as monotherapy or combination therapy. However, we could not collect detailed information regarding antihyperglycemics. Therefore, owing to prescription of various antihyperglycemics and limitation of data retrieval, we are unable to analyze which medications may improve or worsen the survival rates.

**Response to Reviewer #1’s comments:**

1. We have deleted many sentences from the Introduction section in accordance with the reviewer’s advice since they are repeated in the Discussion. We completely agree with the reviewer’s suggestion. Further, we have edited some sentences for better clarity.

We also agree with the reviewer’s concern regarding Line 82. We have included a supplementary table that summarizes the observed findings from references 15, 16, and 21-24 in the Supplementary section.

2. We are grateful for the reviewer’s concern for the potential threat of selection bias in the present study. In the Results section of our manuscript (under the subheading “Baseline characteristics of participants”), the following sentence was written: In total, 2242 lung resections for NSCLC were performed. A total of 23 patients had incomplete data; hence, data of 2219 (99.0%) patients were reviewed in the present study.
3. As the reviewer pointed out, it is better to match exposed (DM) with unexposed (no DM) patients based on confounders such as age, gender, stage, and histological type. We completely agree with the reviewer. In the present study, first, we wanted to grasp the whole picture of individuals with DM and without. Therefore, we analyzed all the patients enrolled and then adjusted the confounders by statistical processing.

One more point the reviewer has raised is that the non-diabetic group included a 17-year-old patient. Checking the raw data, the patient underwent radical resection for NSCLC (adenocarcinoma). The data are correct. However, as pointed out in the section below, we have now presented continuous variables as medians and interquartile ranges (IQRs). Therefore, the number “17” has been deleted from the manuscript.

4. As the reviewer has suggested, we should clarify the timeframe for primary and secondary endpoints. Postoperative complications, as a primary endpoint, was assessed from the end of surgery to discharge from the thoracic surgery unit. Overall survival, as a secondary endpoint, was calculated from the initial event (date of surgery) to the final event (death or loss to follow-up). To clarify the timeframe for the endpoints, we have added a sentence in the Outcome Measures section.

The original grading system for postoperative complications was published by Clavien et al. in 2004. The Japan Clinical Oncology Group (JCOG) has modified and updated the original Clavien-Dindo classification for more precise comparisons of the frequency and severity of postoperative complications among clinical trials across many different surgical fields. As we have cited in the manuscript (reference 25), this grading system for surgical complications has been frequently used and cited in clinical studies. Therefore, we consider this grading system to be valid enough to be used in this clinical study.

Grade was not included in the database. We created corresponding grades for every case, referring to the updated Clavien-Dindo classification.

5. (i) In accordance with the reviewer’s advice, we have changed the description to “presented as medians and interquartile ranges (IQRs).” We have accordingly, we have revised the tables.

(ii) We have added “%” after the actual frequency for all categorical variables.

(iii) The final event to stop the follow-up has been added to the sentence (Line 129 of manuscript with changes highlighted) for a better description of the operational definition of time to event.

(iv) The reviewer pointed out that prediction was not the goal of this study and that the objective was to estimate the relationship between DM and overall survival. We completely agree with the reviewer’s comments, and unfortunately, our description regarding Cox regression was inappropriate. We performed Cox regression to ensure that DM is not an independent risk factor for survival after adjusting for potential confounders. Therefore, we have deleted the following sentence: “For cox regression, initial univariate comparisons were performed using relevant variables and those with an association yielding a p-value of <0.1 were put into the final models.”

Further, we have used upper case “c” in cox.

(v) The comparison of postoperative complications between individuals with and without DM is shown in Table 1. Hence, we would like to leave the number unchanged.

For the primary endpoint (postoperative complications), we have used the adjusted estimates as a binary event and added the adjusted estimates (odds ratios, 95% CI, and p-value) to Table 3.

By creating 4 groups (non-DM [reference], DM with HgA1c% <7%, DM with HgA1c% ≥7% to <8%, and DM with HgA1c% ≥8%), we have also used the adjusted estimates for the primary endpoint (postoperative complications). These data have been added to the manuscript as Table 3.

(vi) The comparison of length of stay between individuals with and without DM is shown in Table 1. Hence, we would like to leave the number unchanged.

The Kaplan-Meier survival curves (Figs 1, 2, 3) have been adjusted for confounders (age and gender).

Table 3 has been renamed as Table 4 because an additional table was inserted after Table 2, which shows Cox regression analysis as a multivariable investigation. For more clarity, we have added “Multivariate” to the table title.

As pointed out by the reviewer, HbA1c% was measured in a very small group of non-DM subjects. We agree with the reviewer’s comment. We have deleted HbA1c% values from Table 4.

6. We have addressed all the points raised by the reviewer and have made the necessary revisions. Fortunately, the revisions have not drastically changed the whole picture. Therefore, we have left the Discussion section unchanged.

Again, we would like to thank the editor and reviewer for the detailed review of our study. All comments have helped us improve the quality of our manuscript.

We await a favorable response and a new evaluation.

Sincerely,

Teruya Komatsu, MD

E-mail: tk.thoracic@gmail.com
